# Frequency Rate of Atelectasis in Patients Following Coronary Artery Bypass Graft and Its Associated Factors at Mazandaran Heart Center in 2013-2014

**DOI:** 10.5539/gjhs.v7n7p97

**Published:** 2015-03-26

**Authors:** Neshat Hasan Niyayeh Saffari, Ebrahim Nasiri, Seyed Nouraddin Mousavinasab, Rahman Ghafari, Aria Soleimani, Ravanbakhsh Esmaeili

**Affiliations:** 1 Student Research Committee, Mazandaran University of Medical Sciences, Sari, Iran; 2 Mazandaran University of Medical Sciences, Sari, Iran; 3 Orthopedic Research Center, Mazandaran University of Medical Sciences, Sari, Iran

**Keywords:** atelectasis, coronary artery bypass surgery, Mazandaran Heart Center

## Abstract

**Introduction:**

As the most common postoperative pulmonary complications after cardiac surgery, atelectasis is one of the most important and serious early postoperative complications and one of the most important causes of prolonged hospitalization, increased costs, and mortality rise. Therefore, the present study was aimed at specifying the frequency of atelectasis in patients following coronary artery bypass graft and its associated factors in Mazandaran Heart Center in 2013-2014.

**Materials:**

The present investigation was a descriptive cross-sectional study in which sequential sampling was used. It was conducted on 375 patients whose coronary artery bypass graft had been performed by the same surgeon and anesthesiologist. For data collection, first the patients’ demographic variables and the information of their surgery were retrieved through their profiles, direct observation, patient inquiry, and the collected data were recorded in the data collection forms. Then, atelectasis was measured before the surgery and on the first and second days after it by taking CXR whose results were checked by two radiologists who were not aware of the previous observations. Data were analyzed through t-test, Pearson test, and Chi-square test using SPSS 16.0.

**Results:**

The results of the present study indicated that, 123 out of 375 patients (32.8%) were diagnosed with at least one type of tattletales during the first three days after the surgery. The mean age of the patients who were diagnosed with atelectasis was 62.9 and most of them were female. The results also showed that there was a significant relationship between postoperative frequency of atelectasis and the patients’ pulmonary diseases and underlying diseases like diabetes and hyperlipidemia, smoking and alcohol use before the surgery, and transfusion of 4 units of packed red blood cells during the surgery (p<0.05).

**Conclusion:**

Atelectasis is the most common postoperative complication which emerges more in patients with pulmonary and underlying diseases than other patients.

## 1. Introduction

Bypass coronary artery surgery is a common way to treat coronary artery blockage. In Iran, 10,000 cases of this surgery are performed every year (Branca et al., 2001). Due to different dominant conditions that are involved with open heart surgery, it is one of the most hazardous surgeries. Most patients experience respiratory problems because of their old age, underlying diseases, sternotomy and its effects on their lung volume and capacity, and pains following sternotomy (Mahoori et al., 2007).

The results of different studies indicated that pulmonary complications are the most important and serious early postoperative problems following cardiac surgery and the most important causes of prolonged hospitalization, increased costs, and mortality rise (Andrejaitiene et al., 2003; SUNAR et al., 2006; Davoudi et al., 2010; Moreno et al., 2011; Doyle, 1999; Loeckinger et al., 2000). Referring to the collapse of small airways and alveoli, atelectasis is the most common of these complications. It can occur in any part of lung but the most involved part is the lower lobe of the left lung and its prevalence has been reported to be 16 to 88 percent (Wynne & Botti, 2004). Atelectasis can occur due to intraoperative causes like prolonged duration of surgery and anesthesia of over 3-4 hours (Wilcox et al., 1988; Brooks-Brunn, 1997), use of thoracic artery (Money et al., 1994), use of cardiopulmonary bypass during surgery and failure to ventilate the patient during pumping and prolonged pump time (Mahoori et al., 2007), and transfusion of 4 units of packed red blood cells during and after the surgery (Goyal et al., 1994; Milot et al., 2001) and postoperative causes like reoperation and the patient’s subsequent anesthesia due to postoperative bleeding or tamponade and placement of stomach catheter (Asimakopoulos et al., 1999; Rodgers et al., 2000). The latter cause can lead to increase in lung compliance and breathing and decrease in the amount of oxygen exchange between the alveoli and the pulmonary artery (Yeager et al., 1987). Diagnosis of atelectasis and how it continues are highly important because it is likely to cause pleural effusion (Davoudi et al., 2010; Gale et al., 1979), prolonged mechanical ventilation, possible pneumonia, and mortality in the patient (Magnusson et al., 1998; Ngaage et al., 2002).

A large number of studies have been conducted on incidence of postoperative pulmonary complications following coronary artery surgery and their causative factors (Wilcox et al., 1988; Brooks-Brunn, 1997; Money et al., 1994; Goyal et al., 1994; Milot et al., 2001; Asimakopoulos et al., 1999; Rodgers et al., 2000). However, since different studies have provided different reports on the frequency of pulmonary complications such as atelectasis and its underlying causes (Harrington et al., 1998; Urschel et al., 1992; Douglas Jr and Spaniol, 2002; Rudra and Sudipta, 2006; Smetana, 2009; Canet & Mazo, 2010; Talavat et al., 2008), no studies have been conducted on the incidence of pulmonary complications in Mazandaran Heart Center despite the fact that it has 10-year experience in performing cardiac surgery, the researchers have served in intensive care unit and have frequently witnessed occurrence of pulmonary complication, continuation of this complication causes the quality of life to drop, hospitalization period to prolong, and costs to rise, and finally the nurses are always present beside the patients and can be of great help if they diagnose the symptoms of pulmonary complications early, the present study was aimed at specifying the incidence of atelectasis and its associated factors among patients who had undergone cardiac surgery during 2013-2014 and had been hospitalized in intensive care unit of Mazandaran Heart Center.

## 2. Materials

The present investigation is a descriptive cross-sectional study that was conducted in order to obtain new data. According to the number of surgeries performed every year in Mazandaran Heart Center (500 cases per year) and with a confidence level of 95% and prevalence rate of 10%, the sample size of 375 patients was determined through sequential sampling method. Patients who due to coronary artery disease and its ischemic events like heart failure in 2013-2014 had undergone coronary artery bypass graft for the first time using cardiopulmonary bypass (CBP) without manipulating cardiac valves by the same surgeon were included in the study. And those patients who had undergone sternotomy and cardiac surgery for the second time for any reason including postoperative bleeding and tamponade were excluded from the study.

The required variables and data were collected based on previous studies (Harrington et al., 1998; Urschel et al., 1992; Douglas Jr and Spaniol, 2002; Rudra and Sudipta, 2006; Smetana, 2009; Canet & Mazo, 2010; Talavat et al., 2008), library studies, and the professors of the department, which were recorded in a data collection form whose reliability had been affirmed by the scientific board of the Nursing and Anesthesia Department of the faculty. The abovementioned form included the patients’ demographic information like age, gender, weight, and height, data on the background of their previous diseases like diabetes and hyperlipidemia and their duration, pulmonary diseases like asthma, chronic pulmonary blockage diseases, and other pulmonary diseases and their duration, preoperative ejection fraction, smoking and alcohol consumption and their duration, background of consuming steroid medicines (the reason for and duration of consumption), and postoperative information collected from the intensive care unit (e.g., duration of patient’s mechanical ventilation during stay in the intensive care unit, the method of extubation of the patient in the intensive care unit, the pre and post operative amounts of O2 saturation, and presence or absence of atelectasis in the patients within three days after the surgery). First, demographic variables and factors related to atelectasis were retrieved from the patients’ profiles, direct observation, and inquiry from the patients by two expert nurses who had at least two years of experience. After initial coordination, they recorded the collected data in the forms. Afterwards, the main variable of the study, i.e. presence or absence of atelectasis, was measured and the results were recorded. In so doing, CXR was taken from the patients before the surgery and within the first and the third days after the surgery (Yánez-Brage et al., 2009) and presence or absence of atelectasis was confirmed by two 5-year experienced radiologists who were unaware of the results of observation and blind to the subject of the study. In cases where the two radiologists had agreement on the results, presence or absence of atelectasis and its intensity were recorded. Kappa coefficient of agreement between the two radiologists was over 80%. Wherever the two radiologists did not agree of the results, a third radiologist was assigned.

According to the radiologists and related references, criteria for measuring the presence of atelectasis included short thin lines in different parts of lung. These lines were divided into three types of linear atelectasis, lobar atelectasis, and segmental atelectasis. Where these lines were observed in different parts of lung and they were not specific to a single part, the atelectasis was linear. When the lines were limited to the fissures between the lung lobes and did not cause the fissures to move, it was called segmental atelectasis, and if the lines caused the fissures to move toward mediastinum, heart, and endotracheal tube, it was considered as lobar atelectasis (Khan et al., 2008). The collected data were fed to SPSS 16.0. To analyze the quantitative data like age, the rate of ejection fraction, etc and compare different groups, T-test was applied. To check the relationship between the tests, Pearson test was applied. And to deal with abnormal situations and compare the qualitative variables, non-parametric tests like Chi-square, Mann Whitney, and Spearman Correlation were applied. And to analyze the data and determine the role of effective factors, unvariate and multivariate regressions were utilized.

## 3. Results

In the present study, out of the 375 patients, 40.5% were female and 59.5% were male. Their mean age was 61.5 years, their mean height was 166.5 cm, their mean weight was 76.14kg, and their mean BMI was 27.5 kg/m^2^.

Hypertension, hyperlipidemia, and diabetes were respectively the most common underlying diseases among the patients. The background of the patients’ underlying diseases is presented in Table 1.

**Table 1:** The frequency distribution of the patients undergoing coronary artery bypass graft based on the background of their underlying diseases in Mazandaran Heart Center in 2013-2014

Disease	N. of patients	%
Asthma	19	5.1%
COPD	18	4.8%
Bronchitis	16	4.3%
Allergies	16	4.3%
Diabetes	136	36.3%
Hypertension	269	71.7%
Hyperlipidemia	208	55.5%
Smoking	89	23.7%
Drug consumption	70	18.7%
Alcohol consumption	8	2.1%
Steroid medicines	9	2.4%

Among allergens, flower pollen with 1.3% has caused the highest level of allergies among the patients who were suffering from allergies.

The results also indicated that a total of 70 patients (18.7%) were drug addicts 65 of whom (17.3%) were consuming opium.

Out of the 375 patients, 270 individuals (72%) had EF≥50% before the surgery and 150 individuals (28%) had EF<50% after the surgery. The results of the study indicated that out of the total of 123 patients who were diagnosed with atelectasis, 44 individuals (41.9%) had EF<50% and 79 individuals (29.3%) had EF≥50%.

Mean duration of surgery was 4.03 hours and mean duration of their CPB was 67.88 minutes.

Table 2 indicates the number of grafts that had been performed on the patients. In most cases (58.1%) leg saphenous vein and thoracic artery were used in performing the graft.

**Table 2:** Frequency of the patients according to the number of performed grafts

N. of grafts	N. of patients	%
2	48	12.8
3	176	46.9
4	130	34.7
5	17	4.5
6	4	1.1
Total	375	100.0

The results of the study indicate that 370 patients (98.7%) were extubated by the nurses and only 5 patients (3.2%) experienced second extubation.

The results also indicated that out of 375 patients, 257 individuals (68.5%) received less than 4 units of packed red blood cells during the surgery and after staying in ICU.

For the main purpose of the study, the results indicated that 24 patients (7.2%) had atelectasis before the surgery and the rate of its occurrence on the first day was 24% (90 cases) 95.6% of which was linear and 4.4% was segmental.

Moreover, it was concluded that on the third day after the surgery, 92 patients (24.8%) had atelectasis 73.1% of which was linear and 26.9% was segmental.

In general, 123 patients (32.8%) had at least one type of atelectasis within three days after coronary artery graft surgery.

The results presented in Table 3 present the percentage of atelectasis occurrence based on the patients’ age, gender, height, weight, and BMI and indicate that there was a significant relationship between age and atelectasis occurrence (p<0.04).

**Table 3:** Frequency distribution of atelectasis based on age, gender, height, weight, and BMI

		Has atelectasis	Doesn’t have atelectasis	P value
Gender	Male	70 (31.4%)	153 (68.6%)	0.48
	Female	53 (9/34%)	99 (1/65%)	
Mean age	9±62.9	60.9±8	0.040	
Mean height	167±6	7±166.2	0.341	
Mean weight	13±77	10±75.5	0.214	
Mean BMI	5±27.7	3±27.3	0.456	

Table 4 indicates the relationship between underlying diseases and occurrence of atelectasis. The results showed that there was a significant relationship between hypertension and smoking and occurrence of postoperative atelectasis.

**Table 4:** The relationship between underlying diseases and atelectasis in patients undergoing coronary artery graft surgery in Mazandaran Heart Center in 2013-2014

Disease	Has atelectasis	Doesn’t have atelectasis	Total	P	
Asthma	Yes	(73.7%) 14	(26.3%) 5	19	<0.001
	No	(30.6%) 109	(69.4%) 247	356	
COPD	Yes	(61.1%) 11	(38.9%) 7	18	0.009
	No	(31.4%) 112	(68.6%) 245	357	
Bronchitis	Yes	(93.8%) 15	(6.3%) 1	16	<0.001
	No	(30.1%) 108	(69.9%) 251	359	
Allergies	Yes	(75%) 12	(25%) 4	16	<0.001
	No	(30.9%) 111	(69.1%) 248	359	
Diabetes	Yes	(44.1%) 60	(55.9%) 76	136	<0.001
	No	(26.4%) 63	(73.6%) 176	239	
Hypertension	Yes	(34.2%) 92	(65.8%) 177	269	0.357
	No	(29.2%) 31	(70.8%) 75	106	
Hyperlipidemia	Yes	(39.4%) 82	(60.6%) 126	208	0.002
	No	(24.6%) 41	(75.4%) 126	167	
Smoking	Yes	(47.2%) 42	(52.8%) 47	89	0.001
	No	(28.3%) 81	(71.7%) 205	286	
Drug	Yes	(37.6%) 27	(61.4%) 43	70	0.254
	No	(31.5%) 96	(68.5%) 209	305	
Alcohol	Yes	(75%) 6	(25%) 2	8	0.010
	No	(31.9%) 117	(68.1%) 250	367	
Steroid medicines	Yes	(100%) 9	(0) 0	9	<0.002
	No	(31.1%) 114	(68.9%) 252	366	
Total number of patients		123	252		

As indicated in Table 4, the results of the study show that there is a significant relationship between the occurrence of atelectasis and underlying pulmonary diseases. Therefore, it can be concluded that patients who had underlying pulmonary diseases like bronchitis (P<0.002), COPD (p=0.009), and allergies (P<0.009) are more likely to have postoperative atelectasis.

Moreover, the results indicated that from underlying diseases like diabetes and hyperlipidemia, with the occurrence of postoperative atelectasis have a significant relationship (P<0.05). Therefore, it can be concluded that patients with diabetes or hyperlipidemia are more likely to face with postoperative atelectasis.

Moreover, as it is indicated in Table 4, patients who had background of smoking, alcohol consumption, and steroid medicines are more likely to encounter postoperative atelectasis.

The results of the study showed that out of 123 patients that had postoperative atelectasis, 44 patients (51.9%) had EF<50% and 79 patients (29.3%) had EF≥50%. Therefore, it can be concluded that there is a significant relationship between postoperative atelectasis occurrence and preoperative EF (p<0.01), so it can be stated that patients who have EF<50% before surgery are more likely to have postoperative atelectasis.

Table 5 indicates the relationship between the occurrence of atelectasis and the type of graft that was performed during the surgery.

**Table 5:** The relationship between the occurrence of atelectasis and the type of graft performed during the surgery

Type of graft	Has atelectasis	Doesn’t have atelectasis
Thoracic artery	0 (0)	(100%) 5
Saphenous vein	(20.4%) 31	(79.6%) 121
Both	(42.2%) 92	(57.8%) 126

The results of the present study indicate that there is a significant relationship between the type of the graft and the occurrence of postoperative atelectasis (p<0.001). As was seen in Table 5, those patients to whom both thoracic artery and saphenous vein were used were more likely to have postoperative atelectasis.

According to the results of the present study, there was no significant relationship between postoperative atelectasis and extubation method.

The results also indicated that out of the 123 patients who had postoperative atelectasis, 66 patients (55.9%) had received 4 or more units of packed red blood cells during surgery and their stay in ICU. Therefore it can be concluded that there is a significant relationship between postoperative atelectasis and the number of packed red blood units during surgery or stay in ICU (p≤0.000). This means that the more number of packed red blood units, will be the higher the risk of postoperative atelectasis.

## 4. Discussion

The results of the study indicated that out of 375 patients, 123 individuals (32.8%) had at least one type of atelectasis within the first three days after the surgery. This finding is in agreement with the results of other studies (Wynne and Botti, 2004) (Wilcox et al., 1988) that indicated 16-88% of the patients face with postoperative atelectasis.

Like other studies conducted by Bazason et al and Walthall et al (Bezanson et al., 2001) (Walthall et al., 2001), the patients mean age was 62.9 and most of them were female. As opposed to gender, there was a significant relationship between postoperative atelectasis and the patients’ age.

The results of the study indicated that preoperative pulmonary diseases like asthma, bronchitis, etc increase the risk of postoperative atelectasis. This finding is in line with the previous studies (Walthall et al., 2001) (Girish et al., 2001). As was presented in Table 4, out of the 14 patients who had asthma and out of 15 patients who had bronchitis, 73.7% and 61.1% had postoperative atelectasis, respectively.

In the present study, as opposed to hypertension that had no relationship with postoperative atelectasis, there was a significant relationship between hyperlipidemia and postoperative atelectasis (p≤0.000) because 44.1% out of 60 patients with diabetes and 40% out of 82 patients with hyperlipidemia faced with postoperative atelectasis. This finding is in line with that of the study conducted by Tanveer et al (Khan et al., 2008).

In the present study, as opposed to previous studies (Khan et al., 2008; Joarder & Crundwell, 2009), there was no significant relationship between drug addiction and postoperative atelectasis, which may be due to the small size of the study sample. However, like other studies (Khan et al., 2008; Joarder & Crundwell, 2009), a great percentage of patients who were smoking and consuming alcohol had postoperative atelectasis. It is interesting that in the present study 100% of the patients who had consumed steroid medicines had postoperative atelectasis.

In the present study, 44 patients (41.9%) who had had EF<50% had postoperative atelectasis.

The results of other present study indicated that thoracic artery graft is one of the factors that cause postoperative atelectasis (Rudra & Sudipta, 2006; Smetana, 2009; Canet and Mazo, 2010). However, in the present study, none of the patients who had used thoracic artery in their graft surgery faced with postoperative atelectasis. And it occurred in patients who in addition thoracic artery in their graft had saphenous vein. The reason for the difference between the present study and other previous studies may be due to other related factors that had been included in the previous studies.

Moreover, like other studies (Rudra & Sudipta, 2006; Canet & Mazo, 2010), in the present study, postoperative atelectasis occurrence was more among patients who had received more than 4 units of packed red blood cells during surgery or stay in ICU. according to the high rate of prevalence of this complication and also some risk factors like underlying pulmonary diseases, diabetes, hyperlipidemia, smoking, alcohol, steroid medicines, EF<50%, etc, it is suggested that the abovementioned points are more taken into consideration before the surgery in order to prevent the confirmed pulmonary complications. It is also recommended that all of the patients who have these preoperative factors should be provided with necessary facilities to be monitored and kept under serious care and after the surgery, special attention should be devoted to intensive nursing care so that the patients can have better respiratory conditions and incidence of atelectasis in them drops. since in the present study it was observed that atelectasis is more likely to occur in patients who had the background of underlying preoperative pulmonary diseases, diabetes, hyperlipidemia, smoking, alcohol consumption, and steroid medicines, it is recommended that further studies should be carried out on the relationship between these factors and postoperative atelectasis.

Moreover, since this study only focused on atelectasis and there are still contradictions among the results of different studies in regard with the incidence of pulmonary complications, it is recommended that a similar study on all postoperative complications be conducted in a wider scope, so that these complications can be prevented by adopting new approaches.

## References

[ref1] Andrejaitiene E. J, Sirvinska S. E, Bolys R (2004). The influence of cardiopulmonary bypass on respiratory dysfunction in early postoperative period. Medicina (Kaunas, Lithuania).

[ref2] Asimakopoulos G, Taylor K. M, Smith P. L, Ratnatunga C. P (1999). Prevalence of acute respiratory distress syndrome after cardiac surgery. Journal of Thoracic and Cardiovascular Surgery.

[ref3] Benzanson J. L, Deaton C, Craver J, Jones E, Guyton R, Weintraub W (2001). Predictors and outcomes associated with early extubation in older adults undergoing coronary artery bypass surgery. American journal of critical care.

[ref4] Branca P, MCgaw P, Light R (2001). Factors associated with prolonged mechanical ventilation following coronary artery bypass surgery. Chest.

[ref5] Brooks-Brunn J. A (1997). Predictors of postoperative pulmonary complications following abdominal surgery. Chest.

[ref6] Canet J, Mazo V (2010). Postoperative pulmonary complications. Minerva anestesiologica.

[ref7] Davoudi M, Farhanchi A, Moradi A, Bakhshaei M. H, Safarpour G (2010). The Effect of Low Tidal Volume Ventilation during Cardiopulmonary Bypass on Postoperative Pulmonary Function. The journal of Tehran Heart Center.

[ref8] Douglas J. M, Spaniol S (2002). Prevention of postoperative pneumothorax in patients undergoing cardiac surgery. American Journal of Surgery.

[ref9] Doyle R. L (1999). Assessing and modifying the risk of postoperative pulmonary complications. Chest.

[ref10] Gale G, Teasdale S, Sanders D, Bradwell P, Russell A, Solaric B, York J (1979). Pulmonary atelectasis and other respiratory complications after cardiopulmonary bypass and investigation of aetiological factors. Canadian Anaesthetists’ Society Journal.

[ref11] Girish M, Trayner E, Dammann O, Pinto-Plata V, Celli B (2001). Symptom-limited stair climbing as a predictor of postoperative cardiopulmonary complications after high-risk surgery. Chest.

[ref12] Goyal V, Pinto R. J, Mukherjee K, Trivedi A, Sharma S, Bhattacharya S (1994). Alteration in pulmonary mechanics after coronary artery bypass surgery: comparison using internal mammary artery and saphenous vein grafts. Indian Heart Journal.

[ref13] Harrington O. B, Duckworth J. K, Starnes C. L, White P, Fleming L, Kritchevsky S. B, Pickering R (1998). Silent aspiration after coronary artery bypass grafting. Annals of Thoracic Surgery.

[ref14] Joarder R, Crundwell N (2009). Chest X-ray in clinical practice, Springer Science & Business Media.

[ref15] Khan T, Chawla G, Daniel R, Swamy M, Dimitri W. R (2008). Is routine chest X-ray following mediastinal drain removal after cardiac surgery useful?. European Journal of Cardio-Thoracic Surgery.

[ref16] Loeckinger A, Kleinsasser A, Lindner K. H, Margreiter J, Keller C, Hoermann C (2000). Continuous positive airway pressure at 10 cm H2O during cardiopulmonary bypass improves postoperative gas exchange. Anesthesia & Analgesia.

[ref17] Magnnusson L, Zemgulis V, Tenling A, Wernlund J, Tyden H, Thelin S, Hedenstierna G (1998). Use of a vital capacity maneuver to prevent atelectasis after cardiopulmonary bypass: An experimental study. Anesthesiology.

[ref18] Mahoori A. R, Nowruzinia S, Farasatkish R, Mollasadeghi G. A, Abbas A (2007). Assessment of the Rapid Shallow Breathing Index as a Predictor of Weaning of Patients with Prolonged Mechanical Ventilation. Tanaffos.

[ref19] Milot J, Perron J, Lacasse Y, Letourneau L, Cartier P. C, Maltais F (2001). Incidence and predictors of ARDS after cardiac surgery. Chest.

[ref20] Money S. R, Rice K, Crockett D, Becker M, Abdoh A, Wisselink W, Kazmier F, Hollier L (1994). Risk of respiratory failure after repair of thoracoabdominal aortic aneurysms. The American Journal of Surgery.

[ref21] Moreno A. M, Castro R, Sorares P, Sant’anna M, Cravo S, Nóbrega A (2011). Longitudinal evaluation the pulmonary function of the pre and postoperative periods in the coronary artery bypass graft surgery of patients treated with a physiotherapy protocol. J Cardiothorac Surg.

[ref22] Ngaage D. L, Martins E, Orkell E, Griffin S, Cale A. R. J, Cowen M. E, Guvendik L (2002). The impact of the duration of mechanical ventilation on the respiratory outcome in smokers undergoing cardiac surgery. Cardiovascular Surgery.

[ref23] Rochelle W, Mari B (2004). Post operative pulmonary dysfunction in adults after cardiac surgery with cardiopulmonary bypass: clinical significance and implications for practice. American journal of critical care.

[ref24] Rodgers A, Walker N, Schug S, MCkee A, Kehlet H, Van Zundert A, MacmahonN S (2000). Reduction of postoperative mortality and morbidity with epidural or spinal anaesthesia: Results from overview of randomised trials. British Medical Journal.

[ref25] Rudra A, Sudipta D (2006). Postoperative pulmonary complications. Indian J Anaesth.

[ref26] Smetana G. W (2009). Postoperative pulmonary complications: An update on risk assessment and reduction. Cleveland Clinic Journal of Medicine.

[ref27] Sunar H, Yuksel M, Salan A, Arar C, Ege T, Ture M, Halici Ü, Duran E (2006). Effect of ventilation on pulmonary epithelial permeability during cardiopulmonary bypass. Koşuyolu Kalp Dergisi.

[ref28] Talavat H, M, Abdollah Panahipour M, Gholamali Molla Sadeghi M, Ghorbanlou M, Fazeli F (2008). Causes of Prolonged mechanical ventilation after coronary artery bypass grafting surgery. strategies. Iranian Heart Journal.

[ref29] Urschel J. D, Parrott J. C. W, Horan T. A, Unruh H. W (1992). Pneumothorax complicating cardiac surgery. Journal of Cardiovascular Surgery.

[ref30] Walthall H, Robson D, Ray S (2001). Do any preoperative variables affect extubation time after coronary artery bypass graft surgery?. Heart & Lung: The Journal of Acute and Critical Care.

[ref31] Wilcox P, Baile E, Hards J (1988). Phrenic nerve function and its relationship to atelectasis after coronary artery bypass surgery. Chest Journal.

[ref32] Yánez-Brage I, Pita-Fernandez S, Juffe-Stein A, Martinez-Gonzalez U, Pertiga-Diaz S, Mauleon-Garcia Á (2009). Respiratory physiotherapy and incidence of pulmonary complications in off-pump coronary artery bypass graft surgery: an observational follow-up study. BMC Pulmonary Medicine.

[ref33] Yeager M. P, Glass D. D, Neff R. K, Brinck-Johnsen T (1987). Epidural anesthesia and analgesia in high-risk surgical patients. Anesthesiology.

